# High Oxygen Modified Atmosphere Packaging Negatively Influences Consumer Acceptability Traits of Pork

**DOI:** 10.3390/foods8110567

**Published:** 2019-11-13

**Authors:** Yunling Peng, Karunia Adhiputra, Anneline Padayachee, Heather Channon, Minh Ha, Robyn Dorothy Warner

**Affiliations:** 1 Faculty of Veterinary and Agricultural Sciences, The University of Melbourne, Parkville 3010, Australia; yunlingp@yahoo.com (Y.P.); k.adhiputra@gmail.com (K.A.); hello@drannelinepadayachee.com (A.P.); minh.ha@unimelb.edu.au (M.H.); 2 Australian Pork Ltd, P.O. Box 4746, Kingston 2604, Australia; heather.channon@australianpork.com.au

**Keywords:** quality, tenderness, oxidation, colour, sensory

## Abstract

Current trends in meat packaging have seen a shift from conventional overwrap to vacuum packing (VAC) and modified atmosphere packaging (MAP). The aim of this experiment was to investigate the effects of high oxygen MAP (HiOxMAP) of pork loins compared with vacuum packed (VAC) on eating quality and colour, after storage in simulated illuminated retail display conditions. Pork loins (*n* = 40) were cut and stored under two packaging methods (HiOxMAP, 80% O_2_, 20% CO_2_; VAC) for up to 14 days, with samples taken at various times for measurements. After 7 days of storage, HiOxMAP samples exhibited inferior consumer acceptability for tenderness, flavor, overall liking, quality and re-purchase intention as well as higher shear force and hardness, relative to VAC samples (*p* < 0.05 for all). Loins stored in HiOxMAP had higher lightness (L*), redness (a*) and yellowness (b*) values at 3 and 7 days, but lower ratio of oxymoglobin to metmyoglobin (oxy/met) values in the meat surface at 14 days of display, relative to VAC samples (*p* < 0.05 for all). The oxy/met ratio declined from 2.3 to 1.7 between days 3 and 14 of display in HiOxMAP samples (*p* < 0.05), whereas the ratio was similar and stayed relatively high for VAC samples. VAC samples produced consistently higher colour values (a*, b*, oxy/met) when left to bloom 30 min after removal from packaging (*p* < 0.05). Lipid oxidation values, measured using thiobarbituric acid reactive substances, in HiOxMAP pork loins, were higher at all time points compared to VAC during the 14 day storage period (*p* < 0.05). The use of vacuum packing for retail shelves, should be considered as the preferred option, over HiOxMAP.

## 1. Introduction

Pork is the one of the most consumed meats and is the most common protein source in the human diet in the world. Meeting the consumers’ demand for high quality pork is a main focus for the pork industry [1] and it has been shown that consumers are increasingly expecting high quality meat [2]. The pork consumers’ purchase intention is negatively affected by inconsistent eating quality [3,4,5]. However, factors such as the supply chain pathways from packaging to retailers to consumers affecting eating quality of pork need to be determined. Understanding the importance of these factors for pork eating quality is essential for the pork industry to improve pork quality and complete an integrated assurance system of pork eating quality [6].

Porks’ presentation is a fundamental gateway between consumers and producers. It has been shown that the consumers’ decision to purchase fresh meat is initially based on the visual qualities, such as colour, marbling and purge loss [7], while sensory attributes including tenderness, juiciness and flavour of meat have been regarded as critical factors for re-purchase intention by consumers [8]. Packaging of pork has been shown to be a factor which could affect pork quality [9,10]. Packaging is not only important to extend meat shelf-life [9], but has also been found to influence eating qualities of meat [9,11]. A number of studies have investigated the impacts that packaging systems have on beef eating quality [12,13,14]. However, the effect of packaging systems on pork eating quality has not been well studied in comparison. MAP with a high oxygen content (70–80%; HiOxMAP) has been shown to improve beef colour, while it negatively affects eating quality of beef [13,15]. In contrast, vacuum packaging, although less desirable in appearance, has been shown to lead to an increase in tenderness and juiciness of beef [13]. A few studies [9,10] have been conducted to examine differential effects of vacuum packaging and HiOxMAP on pork quality. These studies, however, lacked integration of physical measurements with sensory attributes. 

The surface colour of meat is predominantly dependent on the pigment myoglobin (Mb), an iron and oxygen binding protein. Mb incorporates an oxygen atom into its structure when exposed to an oxygen-rich environment. Oxygen-bound Mb (oxymyoglobin) is primarily responsible for the bright cherry-red colour in meat, particularly in the surface of meat in HiOxMAP [16]. In an oxygen-deprived environment, Mb takes the form of an oxygen-free protein deoxymyoglobin and is the prevalent form of myoglobin in vacuum packed meat [17]. When myoglobin or oxymyoglobin, are oxidized and lose an electron, metmyoglobin is formed, which has a brown colour [17]. When metmyoglobin starts to appear in the surface of meat, the meat is taken off the retail shelves, hence measurements of metmyoglobin accumulation in the meat surface over time are used as an indicator of shelf-life [18]. 

As discussed above, although the effects of HiOxMAP and vacuum packaging (VAC) on colour stability and lipid oxidation have been extensively researched for beef [19,20,21], little has been conducted on pork loins, particularly in relation to consumer sensory attributes. As it is well-known that pork is quite different metabolically and biochemically to beef, data on pork is also required. As pork loins are commonly displayed inside HiOxMAP in retail, it is important to determine the full effects of HiOxMAP on the eating quality and shelf quality of pork loins. This study therefore proposed to investigate the effects of pork loin ageing in HiOxMAP or VAC on the texture, colour stability, lipid oxidation, and consumer acceptability and eating quality. 

## 2. Materials and Methods

### 2.1. Experiment Design and Pork Loins

Sensory procedures used in this study were approved by the Human Research Ethics Committee of the University of Melbourne. The experiment was split into two replicates, being two collection days which were two weeks apart. Forty loins from the left and right side of 20 pig carcasses were collected at 1–2 days post-slaughter, from carcasses weighing about 65 kg. Loin samples were placed in a refrigerated trailer at 4 °C and transferred to the meat laboratory in the University of Melbourne.

### 2.2. Sample Cutting and Packaging

Before pork loins arrived at the sensory lab, all the tools and equipment were sterilised with 80% ethanol. Sequentially, two paired loins from the one carcass were transferred from the chiller, 2–3 cm was removed from the posterior end of each loin and discarded. Then, from the anterior end and middle of both loins, three × 12.5 cm sections and five × 4 cm sections were removed and allocated to treatments as shown in Table 1. The allocation allowed for randomization of position between sides and location within the anterior end of the loin. From the posterior end, seven × 2.5 cm sections were removed from both sides and these were allocated to treatments, as shown in Table 1, with randomization of the allocation of samples to treatments according to the position as occurred for the samples for the anterior end. The 4 cm sections were used for objective assessment of Warner-Bratzler shear force (WBSF), compression and cooking loss. These samples were processed on the day of collection (day 0, control) or after the prescribed packaging and ageing period. The 12.5 cm sections were sliced further into 5 × 2.5 cm chops, within each section, and used for sensory assessment. These samples were packaged, subjected to prescribed packaging and ageing and frozen at −20 °C, prior to sensory assessment, with all samples being processed within one month of freezing. The 2.5 cm sections were subjected to treatments shown in Table 1. Colour was measured on samples on day of collection or removal from ageing/packaging treatment. Thiobarbituric acid reactive substances (TBARS) were measured after packaging and freezing of samples at −20 °C, with all samples being processed within one month of freezing.

All the chops (2.5 cm and 4 cm) were first weighed, then packed according to treatment, shown in Table 1. Vacuum packaging was performed on a Multivac C200 (Sepp Haggenmüller GmbH & Co., Wolferschwenden, Germany) using polyamide and polyetheylene vacuum pouches PA/PE 70 (Multivac) with an oxygen permeability less than 65 cc/m^2^ (24 h) and water transmission less than 5 g/m^2^/24 h. HiOxMAP packaging was conducted on a Multivac T200 (Sepp Haggenmüller GmbH & Co., Wolferschwenden, Germany). Packing trays were clear Cryovac (T0D0901C 170 mm × 223 mm, Sealed Air, Australia), a cello soaker pad (130 × 90mm; CBS, Carrum Downs, Australia) was used in all MAP trays and a Biaxially Oriented PolyAmide/Polyethylene/Ethylene vinyl alcohol based film with less than 10 cc/m^2^/24 h and less than 3 g/m^2^/24 h was also used. The gas composition in the MAP was 80% O_2_ and 20% CO_2_. The gas ratio of two packaging packs after ageing treatments were checked by a gas analyser and was 80% O_2_, 20% CO_2_ ± 0.1%. Thiobarbituric acid reactive substances (TBARS) are formed as a by-product of lipid peroxidation (as degradation products of fats), which can be detected by the TBARS assay using thiobarbituric acid as a reagent. The TBARS assay measures malondialdehyde (MDA) present in the sample, as well as malondialdehyde generated from lipid hydroperoxides by the hydrolytic conditions of the reaction [22].

### 2.3. Display and Storage

Control samples for sensory assessment and for objective and chemical measurement were trimmed and packed in vacuum bags on day 0 and stored in a −20 °C freezer. Vacuum packed and HiOxMAP samples were displayed in simulated retail display in a double flat glass door upright refrigerator with LED light (Bromic Refrigeration, Ingleburn, New South Wales, Australia) at 2 ± 1 °C for 3, 7 or 14 days. The samples were rotated between shelves and position daily to minimise the effects of any variation in illumination between shelves. After display, samples for sensory assessment were packed in vacuum bags and stored at −20 °C. Samples for objective measurements were trimmed, then packed in vacuum bags for storage at −20 °C.

### 2.4. Colour Measurement

Instrumental colour analysis on the surface of meat samples was conducted using a Hunterlab Miniscan EZ (Hunter Assoc. Labs Inc., Virginia, VA, USA) that had been calibrated against white and black reference tiles. The settings used for colour measurement were D65 illuminant and 10° observer angle. CIE L* (lightness), a* (redness) and b* (yellowness) values [23] were obtained from the average value of two readings on the surface of loin samples. Control samples were measured prior to packaging and after a 30 min bloom at 4 °C. HiOxMAP treated samples were measured as soon as the pack was opened, at the completion of ageing/display. VAC packaged meat was measured as soon as the meat was opened (‘unbloomed’) and also after 30 min blooming (‘bloomed’) at a low temperature (4 °C) in the refrigerator. The Hunterlab Miniscan also gives percentage reflectance across the wavelengths of light from 400 nm to 700 nm. The ratio of oxymyoglobin:metmyoglobin (oxy/met) was calculated by dividing the percentage of light reflectance at wavelength 630 nm by the percentage of light reflectance at wavelength 580 nm as recommended by Hunt and King [17] and used in previous studies [18,24,25,26].

### 2.5. Objective Measurements of Texture and Water Loss

For purge loss, samples from 3 and 7 days in VAC or HiOxMAP were weighed before and after packaging. The purge loss was calculated as the initial weight minus the final weight, divided by the initial weight, multiplied by 100 to obtain percentage as described previously [27].

Cooking of pork samples was performed according to procedures outlined in [28], with modifications. Pork samples were prepared into 2 × 70 g ± 5 g blocks to be used for either Warner-Bratzler Shear Force (WBSF) or compression assessment. For WBSF and compression, meat samples were placed in individual plastic bags and suspended in a preheated 70 °C water bath (F38-ME, Julabo, 77,960 Seelbach, Germany) and cooked until an internal temperature of 70 °C was reached. Internal temperature was monitored using temperature probes inserted into the core of the sample (Grant Instruments, Cambridge, United Kingdom). After cooking, samples were cooled in iced water for 30 min. Samples were then dried with paper towel and weighed. After weighing, samples were placed on a tray and covered by plastic wrap to minimise moisture loss, and stored at 4 °C overnight in preparation for WBSF and compression assessments. Cooking loss is calculated as the initial weight minus the final weight, divided by the initial weight, multiplied by 100 to obtain percentage.

Objective texture measurements were conducted, using the Warner-Bratzler shear force (WBSF) method of [29] with modifications [28], on 0, 3 and 7 day samples after completion of treatments. For each sample, six 1 cm^2^ × 4–5 cm rectangular strips were cut parallel to the direction of muscle fibres, and the WBSF was measured using a Warner-Bratzler shear force blade (V-shaped) adapted to a Lloyd machine (Lloyd Instruments Ltd, Largo, FL, USA) with a 500 N load cell, and the shearing speed was set at 300 mm/min. The peak of the shear force was recorded and the mean was calculated from the 6 sub-samples.

A modified compression method was used to analyse the effects of connective tissue on tenderness of meat using methods of [30] with modifications described by [28]. A 0.63 cm diameter flat-ended probe was attached to the Lloyd Machine and a 500 N load cell was used. The procedure included 2 penetrations. Firstly, the probe was driven vertically down at a speed of 50 mm/min into 80% of the thickness of a 1 cm thick sample. The muscle fibres were horizontal to the placement of the sample on the plate and perpendicular to the probe. The force work was measured, which was called initial penetration. Secondly, the probe was raised and then lowered, to penetrate the sample a second time. The force work was recorded as second penetration. The parameters measured from compression were:

- Hardness: the force work required for the initial penetration,
- Cohesiveness (or called ease of break down): the reduced force work required for second penetration from work needed for the initial penetration,
- Chewiness: the force required to achieve hardness and cohesiveness.

### 2.6. TBARS Analysis

TBARS analysis was conducted using a modified extraction method of Witte, et al. [31]. 20 g duplicate samples of pork were homogenized with 50 mL 20% trichloroacetic acid in 2 M phosphoric acid in a Nutribullet Pro 900 (Nutriliving, Pacoima, CA, USA) for 50 s. The homogenate was then diluted with 50 mL deionised water and blended for another 15 s. 50 mL of the homogenate was filtered through Whatman no.1 filter paper. 4mL of the filtrate was transferred to a test tube in duplicate followed by the addition of 4 mL of 2-thiobarbituric acid (5 mM). The test tube was stoppered and kept in the dark for 15 h at room temperature with appropriate standard solutions using 1, 1, 3, 3 tetraethoxypropane as standard. The resulting colour was measured at 532 nm in a spectrophotometer, and absorbance converted to malonaldehyde mg/kg of meat. 

### 2.7. Sensory Assessment 

The design of this sensory assessment was in accordance with previous sensory studies on pork [28] but with higher numbers of consumers and samples. These procedures used, including allocation, cooking and serving, have previously been used in research in beef, pork and lamb [27,28,32,33] and are described in Watson, et al. [34] with modifications to the cooking protocol for pork described in Channon, Taverner, D‘Souza and Warner [28]. Consumer panelists aged between 18- and 60-years-old were recruited from students and staff of the University of Melbourne. Consumers were asked to participate in a one-hour sensory panel consisting of 10 panelists. Five consumer panel sessions were conducted with samples for cooking and consumption from each carcass and treatment within a carcass, allocated evenly across the five sessions. The total number of consumers was 50, and the total number of samples was 300, from 20 carcasses. Each consumer consumed six pieces of meat, 60 samples were served and consumed in a session, and no consumers were used in more than one session. 

The allocation of samples within one sensory session was randomized based on 10 consumers consuming pieces of meat from 10 carcasses, and was designed by using Latin–square design. Every consumer tasted the three treatments twice, enabling a high degree of comparison between treatments and between carcasses. Serving orders can be a factor in influencing sensory results and therefore random serving orders were used in the sensory evaluation. The randomisation of samples presented to consumers was also designed based on Latin-square design. 

Allocation of frozen samples into sensory sessions was achieved by sorting samples in the sensory lab three days before sensory sessions. Samples were taken out from freezer and laid out on tables. Using an allocation sheet, samples were sorted into different sessions with 30 steaks in each session, then placed back in the freezer. Before thawing, carton numbers and samples in cartons were checked to avoid errors and also samples were placed in a single layer to ensure consistent rates of sample thawing. Samples were thawed in a refrigerator at 2 ± 1 °C for 24 h before cooking. Prior to cooking, the temperature of steaks were recorded and ranged between 5 and 8 °C. After thawing, samples were taken out from vacuum bags ready for cooking.

Cooking was conducted on two Silex grillers (S-161 Silex Elektrogerate GmbH, 22143 Hamburg, Germany). Five steaks were cooked at a time on each Silex griller, thus 10 samples were served simultaneously to 10 consumers. As outlined by Channon, Taverner, D‘Souza and Warner [28], pork samples were cooked at 160 °C for 3 min 10 s until a medium level of doneness was achieved. After two min resting, the internal temperature measured was 70 °C, consistent with a pilot reference study conducted. After cooking, samples were placed on a cutting board and the 4 edges were removed from samples with the middle part used for consumption. Each consumer was presented with 6 cooked samples over a 35 min period. 

In each session, consumers were seated in individual sensory booths. Before evaluation, consumers were given instruction and explanations and were asked to fill out a questionnaire to record the demographic information including name, age group, and pork consumption habit. 

Each consumer was asked to assess each sample for aroma (dislike extremely to like extremely), tenderness (not tender to very tender), juiciness (not juicy to very juicy), flavour (dislike extremely to like extremely), overall liking (dislike extremely to like extremely) and quality grade (unsatisfactory, average, premium) and purchase intention (not buy it, might buy it, will buy it) on a 100 mm line scale with 0 representing the minimum and 100 representing the maximum. In the case of quality grade and purchase intention, they were anchored with words at either end of the scale, as for the other traits, but also had the words ‘average’, and ‘might buy it’ in the middle of the scale. The 10 tastings for each sample were averaged to give the final eating quality score for each. The methodology for sample collection, allocation to treatments, allocation to consumer sessions, cooking, running of consumer sessions, analysis of the line scales, and averaging were first described in Watson, Gee, Polkinghorne and Porter [34], and have been used in consumer research on beef [32,33,35], pork [28] and lamb [27] in Australia and in many countries including Japan [36], South Africa [37] and Korea [38].

### 2.8. Statistical Analysis 

The GENSTAT program (Version 16, release 16.1.0.10916, 64-bit) was used to do an analysis of variance to determine the effects of packaging and ageing on the texture and the sensory attributes of the pork loin samples. Block factors of carcass and replicate were used in ANOVA for purge loss, cooking loss, WBSF, hardness, chewiness, cohesiveness, TBARS and colour measurements. The analysis of variance for sensory attributes included blocking factors of carcass, replicate and session in the ANOVA. 

## 3. Results

### 3.1. Sensory Assessment 

**Figure 1:**
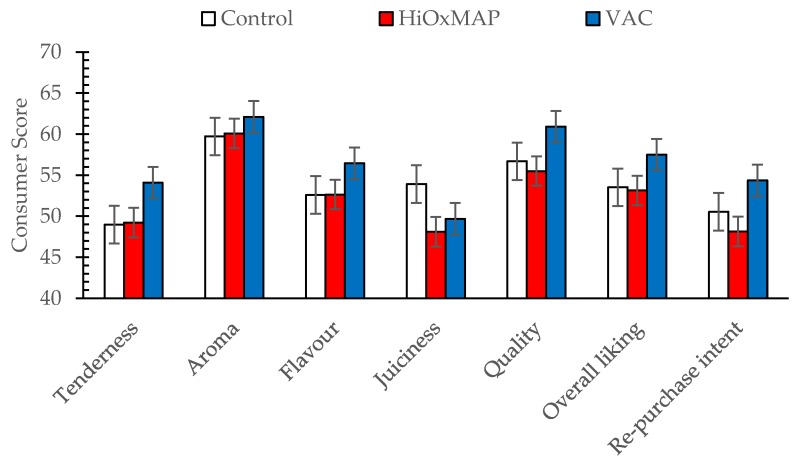
Effects of packaging (control, no packaging; HiOxMAP, Modified Atmosphere Packaging in 80% O_2_, 20% CO_2_ for 7 days; VAC, vacuum packaged for 7 days) on consumer scores for sensory attributes of porcine longissimus. Data are presented as least squares means ± s.e.d. Tenderness, *p* < 0.05; Aroma, *p* > 0.05; Flavour, *p* < 0.10; Juiciness, *p* < 0.05; Quality grade, *p* < 0.05; Overall liking, *p* < 0.05; Re-purchase intent, *p* < 0.05.

The impact of packaging and ageing on sensory attributes of pork are shown in Figure 1. Samples vacuum packed for 7 days had higher sensory scores, and hence were preferred, for tenderness, flavour, overall liking, and quality relative to control and 7 day HiOxMAP samples (*p* < 0.05 for all except flavour, *p* < 0.10). In addition, the 7 day VAC samples had higher scores for re-purchase intention, relative to the 7 day HiOxMAP samples (*p* < 0.05). In terms of objectively measured tenderness, vacuum packaging of pork resulted in an average WBSF value of 54.1N, which were approximately 5 units higher than the control and HiOxMAP treated samples (see below for further results for WBSF). Generally, meat must have a PSF of <40 N (4.1 kg) in order to ensure high levels of consumer acceptability [39]. 

Packaging and ageing treatments did not result in a change in sensory scores for aroma of pork in the current study. This is in agreement with a study by Lund, Lametsch, Hviid, Jensen and Skibsted [10] who examined the effect of vacuum and HiOxMAP on pork aroma and flavour attributes using a trained sensory panel (not consumer panel). However, vacuum packaging increased tenderness scores. Attributes of VAC samples for overall like, quality grade and purchase intention corresponded with tenderness scores. Channon, et al. [40] concluded that tenderness of pork aged 7 days in vacuum bags improved by 4.7 units relative to 2 day aged pork samples and the overall liking score also improved by 3.0 units. The results presented in this study were also consistent with those from Jonsäll, Johansson and Lundström [7], where pork aged 8 days was more tender than 4 day aged pork. In terms of packaging, HiOxMAP negatively impacted tenderness in comparison with VAC, which agreed with findings in a previous study [10]. In addition, HiOxMAP treatment of pork resulted in a similar sensory score to that of the control samples. For the attribute of juiciness, both VAC and HiOxMAP samples had lower scores than the control samples, which may be explained by the higher purge loss. 

### 3.2. WBSF and Compression 

Both packaging and ageing influenced the tenderness of pork (*p* < 0.01), as presented in Table 2. WBSF was highest in control samples, intermediate for HiOxMAP samples at 3 and 7 days, and lowest for VAC samples at both 3 and 7 days. The WBSF value of control samples was 29.2 N, samples stored in VAC for 3 days had a WBSF of 20.1 N, and after 7 days ageing, the value was further reduced to 17.9 N. Pork aged in HiOxMAP either for 3 or 7 days had a similar WBSF of about 22.5 N, which supports the reduced sensory scores for tenderness for HiOxMAP samples.

There were effects of packaging type and ageing period on the hardness of pork loins (*p* < 0.001) as presented in Table 2. Control and HiOxMAP samples all had similar hardness whereas VAC samples at 3 and 7 days had lower hardness values, which supports the reduced sensory scores for tenderness for HiOxMAP samples. The effects of packaging and ageing on cohesiveness are shown in Table 2, and there was an interaction between packaging and ageing (*p* < 0.05) such that for HiOxMAP, cohesiveness increased between days 3 and 7 whereas there was no change for VAC samples. The influence of packaging and ageing on chewiness is also shown in Table 2 and there was an effect of packaging; VAC samples had lower chewiness relative to HiOxMAP (*p* < 0.05).

The physical forces exerted on meat during masticationg are shear force, compression (hardness, chewiness and cohesiveness) and tensile force. These mechanical forces are defined as objective assessments in estimating the tenderness of meat [41]. Data presented in this report indicate that packaging and ageing affected shear force and hardness, and to a lesser extent cohesive and chewiness. VAC samples showed a decrease in shear force values at days 3 and 7 day of ageing. Channon, Kerr and Walker [40] found that pork aged for 7 days in VAC resulted in a reduction in WBSF of pork in comparison with 2 day post-slaughter ageing. In addition, similar results were obtained for beef [15]. In this study, pork aged in HiOxMAP presented a lower shear force than control samples, although not as low as VAC samples, likely because there was initially some tenderization in HiOxMAP, then oxidation of calpain in the high oxygen environment prevented further tenderization and proteolysis [42]. This is similar to our previous results in lamb where sensory (WBSF not measured) tenderness increased in vacuum packed samples between 5 and 10 days of storage, but the tenderness of HiOxMAP samples decreased over the same period [42]. This also corresponded with results of a previous study [10] which indicated HiOxMAP inhibited the tenderization of pork. Sorheim, et al. [43] also indicated that HiOxMAP did not result in a decrease in the shear force value of beef stored up to 14 days, hence ageing was inhibited also in their study. Therefore, from an objective physical measurement perspective, ageing in vacuum packaging resulted in more tender meat in comparison with ageing in HiOxMAP.

### 3.3. Purge Loss 

**Figure 2:**
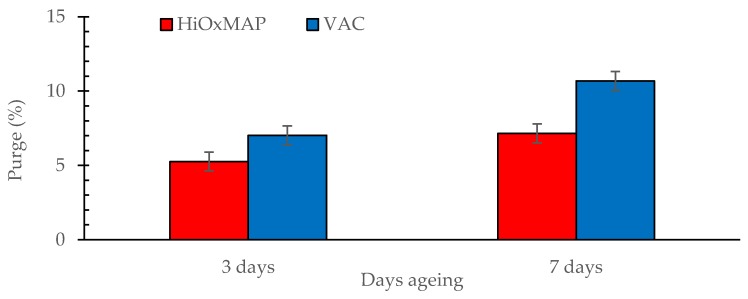
Effect of packaging (HiOxMAP, high oxygen modified atmosphere packaging, 80% O_2_, 20% CO_2_; VAC, vacuum packaging) and ageing (3 or 7 days) on purge loss. Data are presented as least squares means ± s.e.d for the interaction. Packaging, *p* < 0.001; Ageing, *p* < 0.001; Packaging.Ageing, *p* < 0.10).

The influence of packaging and ageing on purge loss is presented in Figure 2. Purge loss was higher in VAC stored pork loin than HiOxMAP and was also higher for 7 days aged samples relative to 3 days aged samples (*p* < 0.001 for both). Also, there was a trend for an interaction between packaging and ageing (*p* < 0.10). Over the ageing period from 3 to 7 days ageing, purge loss of VAC pork increased from 6.8% to 10.5%, while purge loss of pork in HiOxMAP only slightly increased from 5.1% to 7.0%. Purge loss is associated with fluid loss from meat during storage [13], and is an indicator of the moisture content lost from the pork. This has been shown to mainly be associated with juiciness, tenderness and flavour of meat. Higher purge loss was observed in pork aged in VAC than in pork stored in HiOxMAP, which was consistent with a study by [13] who showed that the amount of purge loss of beef stored in vacuum was higher compared to beef stored in HiOxMAP. The increase in purge loss with an increased ageing time also agreed with other results [13]. The high weight loss in vacuum packed samples could be explained by fluid loss under the influence of vacuum pressure [13]. However, these results were contradictory with those of a pork study in which pork aged in HiOxMAP was found to have a greater purge loss than pork stored in vacuum condition, and the purge loss increased significantly with the increased ageing time from 3–7 days [8,10]. In 2009, Nam et al. [8] reported that pork with a low purge loss during storage has been shown to be more tender and have a better flavour. Interestingly, in the current study, HiOxMAP samples, which appeared to have a lower purge loss received a lower score for tenderness and flavour. In terms of juiciness, VAC stored pork with higher purge loss had a higher score than HiOxMAP, while control samples with no purge loss showed highest score on attribute of juiciness. These results in combination indicate that purge loss in this study could not be used as an independent variable to explain the effect of packaging and ageing on pork eating quality. 

### 3.4. Cooking Loss

Differences were observed in cooking loss among packaging × ageing treatments as displayed in Table 2 (*p* < 0.05). Cooking loss for control and pork loin steaks aged in HiOxMAP for 3 days were the highest, HiOxMAP and VAC samples stored for 7 days were intermediate and 3 day VAC samples had the lowest cook loss. Cooking loss is defined as the loss of fluid, along with soluble substances, from meat during the cooking process, and it has been established that it could affect tenderness and juiciness [44]. In this study, a lower cooking loss was observed for pork stored in vacuum packaging in comparison with HiOxMAP and control samples. The reduction in cooking loss of vacuum packed samples was correlated with both a lower shear force and a higher sensory score for tenderness of vacuum packed pork. It has been previously noted that panelists do not prefer pork which had high cooking loss and it was regarded as tough pork [8]. Lagerstedt et al. [13] found that beef aged for 7 days in HiOxMAP had a higher cooking loss than beef aged in vacuum bags. Although the control samples in the current study had a higher cooking loss, a higher sensory score was given for the juiciness attribute of the control samples, compared with pork stored in vacuum and HiOxMAP. A possible explanation for the control samples attracting a higher sensory score for juiciness is the lower purge.

### 3.5. Colour

**Figure 3:**
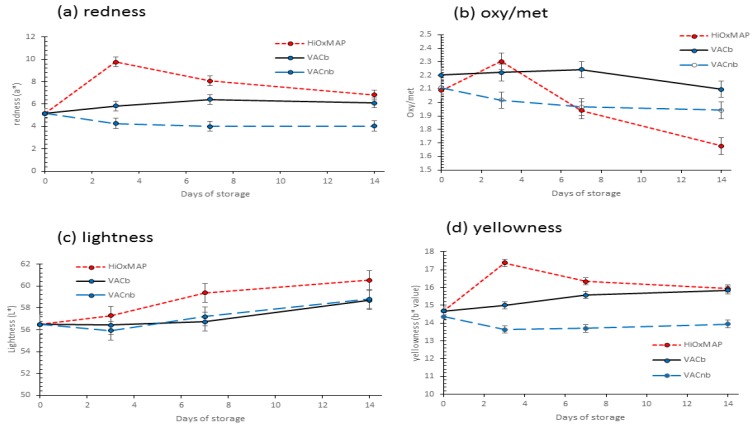
Effect of packaging (HiOxMAP, high oxygen modified atmosphere packaging vs VACnb, vacuum packing, colour measured after no bloom vs VACb, vacuum packing, colour measured after 30 min bloom) and day of storage (0, 3, 7, 14 days) on (**a**) the surface redness (a*), Packaging, Ageing, Packaging.Ageing, *p* < 0.001 for all, (**b**) oxy/met (R630/R580) values, Packaging, Ageing, Packaging.Ageing, *p* < 0.001 for all, (**c**) lightness (L*), Packaging, *p* < 0.001; Ageing, *p* < 0.001; Packaging.Ageing, *p* > 0.05 and (**d**) yellowness (b*), Packaging, Ageing, Packaging.Ageing, *p* < 0.001 for all. Each point is a least squares mean ± s.e.d. for the interaction.

The effect of time and packaging on L*a*b* values and oxy/met ratio is given in Figure 3. The L* values of HiOxMAP, VACb (vacuum packed pork colour after opening pack and blooming for 30 min) and VACnb (vacuum packed pork colour after opening pack and immediate colour measurement, no blooming) were not different after 3 days of storage (*p* > 0.05). After prolonged storage, HiOxMAP packaging produced the palest colour samples and this was significantly different after 7 and 14 days of storage (*p* < 0.05). An increase in lightness is attributed to relative contents of chemical forms of myoglobin and increased light scattering due to protein denaturation [23]. The increased lightness in HiOxMAP meat during storage indicates that HiOxMAP meat loses contrast over time, producing less pink colour in the meat [45].

The a* values of pork loins were highest at 3 days of storage when packed in HiOxMAP (*p* < 0.001), but gradually fell as storage time progressed. The a* values of HiOxMAP and VACb after 14 days of storage were not significantly different (*p* > 0.05). However, HiOxMAP loins still produced higher redness compared to VACnb after 14 days of storage (*p* < 0.001). Redness level is an important factor in producing the desired pink colour in pork [46,47]. 

The b* values of pork loins followed a similar trend to a* values, where HiOxMAP loins produced the highest b* values after 3 days of storage (*p* < 0.0001), but gradually decreased as storage time progresses. The b* values of VACb and HiOxMAP at day 14 were not different (*p* > 0.05) but VACnb and HiOxMAP were different (*p* < 0.001). O’Sullivan, Byrne, Martens, Gidskehaug, Andersen and Martens [46] and Chizzolini, et al. [48] indicated that b* values were important in determining the onset of brown pigmentation, and that unsatisfactory appearance in meat were associated with a more pronounced yellow tint which largely depends on the relative balance of a* and b*. 

For the oxy/met ratio, HiOxMAP produced the highest value after 3 days of storage (*p* < 0.05) but declined after this time period. At 7 days storage, the oxy/met ratio of HiOxMAP meat was lower than VACb, and was lower than both VACb and VACnb after 14 days of storage (*p* < 0.001). The oxy/met ratio is measured from reflectance and is a measurement which closely relates to what the human eye and brain can see [23]. This value gives an indication of changes in the predominant pigment of myoglobin that is present on the surface of meat—particularly oxymyoglobin (responsible for the red colour) and metmyoglobin (responsible for the brown colour). A reduction in oxy/met ratio is correlated to browning of meat, as prolonged exposure of air as well as other oxidation mechanisms promotes oxidation of oxymyoglobin to metmyoglobin [17]. The Australian lamb industry specifies that if the oxy/met ratio is < 3, the meat would be unacceptable to consumers [49]. According to our results, pork has lower oxy/met ratios than lamb and thus the cutoff for acceptability is yet to be established for pork. But it can certainly be concluded that the pork packaged under HiOxMAP had inferior colour relative to vacuum packed meat after 7–14 days of storage. 

Brewer and McKeith [45] suggested that categorising meat colour on the basis of L*, a* and b* values to varying degrees of pink colour is possible, and many studies have suggested that meat quality can be attributed to colour measurements [46,48,50,51]. The combination of L*a*b* measurements to categorise meat colour seems to have strong interactions with one another – with each colour attribute making a contribution to the overall appearance quality of pork. However, pork meat which is generally considered to be very light pink and pale in appearance is associated with high L*, low a* and high b* values, whilst pork meat which is dark pink is associated with low L*, high a* and high b* values [45].

The results have indicated that HiOxMAP packaging caused an increased rate of decline in the oxy/met ratio on the surface of pork loins, indicating that metmyoglobin formation is accelerated in a high oxygen environment which produced the undesirable pale, brown colour. Blooming of VAC loins seemed to have a significant effect in increasing oxy/met ratio compared to no blooming (*p* < 0.001), suggesting the beneficial effects of VAC and 30 min blooming on minimising metmyoglobin formation and subsequently general appearance of pork loins in long term storage. 

The high L* and low a* values that were measured on HiOxMAP loins after 14 days of storage indicate that the colour of the steak is very light pink in colour, and quite pale compared to its colour measurements after 3 days of storage. Colour measurement values are even tending towards colours that can be seen in PSE (pale, soft, exudative) pork —which is described as L* > 57 [48]. This gives further insight that VACb or VACnb are the best methods of maintaining colour for long term storage of meat. However, as this is a method of storage, which requires the removal of meat from the packaging, it will require an extra step of intervention by removing the meat and placing it at a low temperature in a different atmosphere; this requires extra labour and time, and is not desirable for industry compared to HiOxMAP packaging, which are packed in the format purchased by the consumers. Thus, it is recommended that vacuum skin packing should be considered, which would be equivalent to VACnb.

### 3.6. TBARS 

**Figure 4:**
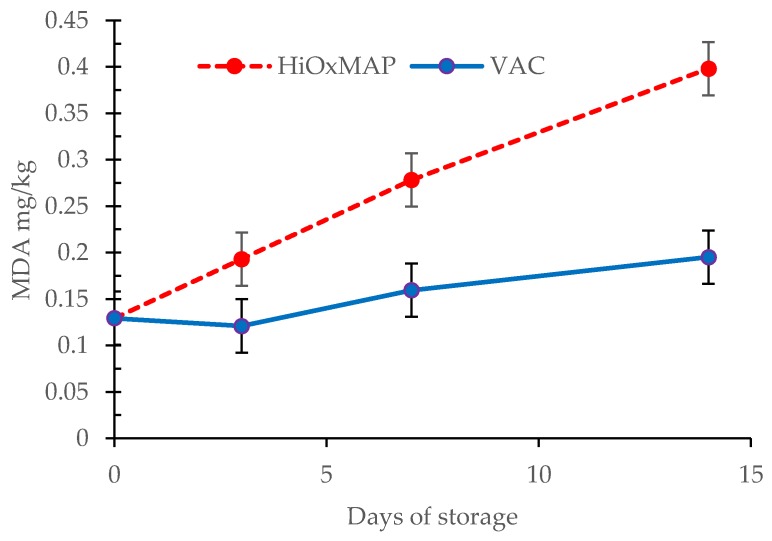
Effect of packaging (HiOXMAP, high oxygen modified atmosphere packaging vs VAC, vacuum packing) and day of storage (0, 3, 7, 14 days) on TBARS (MDA, Malonaldehyde, mg/kg of meat) values. Each point is a least squares mean ± s.e.d for the interaction. Packaging, *p* < 0.001; Ageing, *p* < 0.001; Packaging.Ageing, *p* < 0.001).

Figure 4 displays the effects of VAC and HiOxMAP on TBARS values after simulayed retail storage. There was an interaction between packaging and ageing as well as main effects of packaging and ageing (*p* < 0.001 for all). HiOxMAP had a significant impact on lipid oxidation development, with TBARS values being higher at 3, 7 and 14 days of storage (*p* < 0.001) relative to VAC. VAC samples did not show an overall increase in TBARS values—with day 0 samples and day 14 samples being similar (*p* > 0.05) and were consistent with previous studies [9,52,53]. This indicates that vacuum stored pork loins had minimal lipid oxidation, i.e., almost zero development of oxidation products during 14 days of storage. On the other hand, increases in lipid oxidation in HiOxMAP samples occurred with an increase of MDA mg/kg from 0.12 to 0.37 mg/kg—almost a three-fold increase during the 14-day period storage. Lipid oxidation is therefore strongly affected by HiOxMAP and high oxygen environments. 

Lipid oxidation is closely correlated to pigment oxidation due to production of free radicals and ROS [54], and the results are consistent with these findings. Lipid oxidation promotes myoglobin oxidation [55] and decreases of surface redness as well as a decrease in oxy/met ratio indicates that as lipid oxidation progresses in HiOxMAP product, metmyoglobin production also increased. 

Rancidity is described as a flavour, which develops due to various factors that create undesirable and unacceptable characteristics in meat. The connection of lipid oxidation and rancidity has not been fully established. Campo, et al. [56] attempted to create a link of TBARS values with sensory qualities of rancidity in beef, and found that the point of TBARS value of 2 is a limiting point for where rancid flavour overpowers beef flavour. The results largely varied, however, due to various limiting factors such as personal thresholds and experience of panellists. Furthermore, various other VOCs (volatile odour compounds), which are produced by other mechanisms other than lipid oxidation are also involved in creating the general rancid flavour in pork [57]. According to Greene and Cumuze [58], the TBARS value at which rancid odour is first observed by panelists is 0.5 to 1.0 mg MDA/kg but malonaldehyde content contributes only to a small part of the total odour complex. 

The values of TBARS found in this study have indicated that malonaldehyde production in HiOxMAP and VAC meat did not reach unacceptable levels of lipid oxidation—with the largest TBARS value being 0.37 MDA mg/kg. However, the elevated rate of lipid oxidation found in HiOxMAP product provides further explanation of the deterioration of colour properties, as well as indicating that shelf life qualities were negatively impacted compared to VAC packaging. 

## 4. Summary and Recommendations

Pork loin samples subjected to HiOxMAP exhibited inferior consumer acceptability for tenderness, flavour, overall liking, quality and re-purchase intention relative to samples vacuum packed for 7 days. Samples vacuum packed for 7 days also had lower shear force and hardness values compared to samples undergoing HiOxMAP for 7 days. In addition, HiOxMAP maintained a high a* value in samples during 3 days of storage and higher L* value at the end of storage. Blooming of vacuum packaged meat after long term storage resulted in greater colour stability, as shown by the same a* values, lower L* values and less browning (higher oxy/met) compared to HiOxMAP packaging. HiOxMAP packaging resulted in higher rates of lipid oxidation compared to VAC, which indicated a reduction in shelf life quality. 

This research, based on a small number of samples, has shown that pork loins have lower ‘colour and oxidation’ shelf life when packed in HiOxMAP compared to vacuum packed samples. The results have also shown that pork loins stored in HiOxMAP have elevated lipid oxidation, reduced contrast in colour and increased browning. The findings suggest that pork loins are suited for packaging in HiOxMAP for less than 7 days, possibly only 3 days, and VAC packaging should be considered for storage for any time period longer than 3–7 days. Hence, the use of vacuum packing of pork for retail shelves should be considered as the preferred option, over HiOxMAP.

Using similar packaging and consumer sensory methodology, it has been shown that beef and sheep meat packed in HiOxMAP have reduced consumer sensory scores of 10–12 for beef [59] and 10–15 for sheep meat [42], over 9–10 days of retail display. The packaging of pork in HiOxMAP in our study resulted in a reduction of 5–6 sensory scores over 7 days, which although lower than seen in beef and pork, was over a shorter period. Hence, it is evident that in order for the pork industry to accurately and effectively manage stock and supply within the supply chain to ensure that high quality pork is delivered to the consumers, the effect of HiOxMAP on eating quality is an important consideration. In order to extend the results to the wider industry, more research is required on a greater range of muscles and under a wider range of conditions. 

## Figures and Tables

**Table 1:** Allocation of samples within the length of the striploin, to measurements and treatments across 0, 3, 7, and 14 days of ageing.

Position in Both Loins	0 Days	3 Days VAC ^1^ & HiOxMAP ^1^	7 Days VAC & HiOxMAP	14 Days VAC & HiOxMAP
Anterior section 5 × 4 cm	Warner-Bratzler, Compression, cooking loss	Warner-Bratzler, Compression, cooking loss	Warner-Bratzler, Compression, cooking loss	
Middle section 3 × 12.5 cm	Consumer sensory		Consumer sensory	
Posterior 7 × 2.5 cm	Colour, TBARS	Colour, TBARS	Colour, TBARS	Colour, TBARS

^1^ VAC, vacuum packaging; MAP, high oxygen modified atmosphere packaging (80% O_2_, 20% CO_2_).

**Table 2:** Effect of packaging (control, no packaging; HiOxMAP, high oxygen modified atmosphere packaging, 80% O_2_, 20% CO_2_; Vacuum, vacuum packaging) and ageing (0, 3, 7 days) on objective meat quality traits of pork loin. Data are presented as least squares means.

Treatment	Control	HiOxMAP		Vacuum		SED ^1^	F-values			
Days Ageing	0 Days	3 Days	7 Days	3 Days	7 Days		Control	Pack	Age	Pack.Age
Cook loss (%)	19.28	19.54	18.97	16.73	18.45	0.590	<0.001	<0.001	0.337	0.017
Chewiness	10.00	10.12	10.29	9.74	9.15	0.452	0.111	0.021	0.505	0.238
Cohesiveness	0.435	0.412	0.436	0.427	0.421	0.0076	0.14	0.515	0.362	0.043
Hardness	34.18	34.53	34.82	31.59	30.65	0.874	0.001	<0.001	0.599	0.329
WBSF (N)	29.23	21.56	22.39	20.23	17.94	0.973	<0.001	<0.001	0.292	0.027

^1^ SED for the interaction term.
